# Electronic Cigarette and Traditional Cigarette Use among Middle and High School Students in Florida, 2011–2014

**DOI:** 10.1371/journal.pone.0124385

**Published:** 2015-05-13

**Authors:** Lauren Porter, Jennifer Duke, Meredith Hennon, David Dekevich, Erik Crankshaw, Ghada Homsi, Matthew Farrelly

**Affiliations:** 1 Florida Department of Health, Tallahassee, Florida, United States of America; 2 Public Health Research Division, RTI International, Research Triangle Park, North Carolina, United States of America; University College London, UNITED KINGDOM

## Abstract

Recent youth trends in the prevalence of e-cigarette and traditional cigarette use in Florida were examined in a cross-sectional, representative state sample from 2011 to 2014. Traditional cigarette use among youth declined during the study period. Experimentation with and past 30-day use of e-cigarettes among Florida youth tripled over 4 years. Past 30-day e-cigarette use exceeded traditional cigarette use in 2014; 10.8% of high school and 4.0% of middle school students reported recent e-cigarette use, compared with 8.7% of high school and 2.9% of middle school students for traditional cigarettes (*P*<0.001). By 2014, 20.5% of high school and 8.5% of middle school students reported ever use of e-cigarettes. Among ever e-cigarette users in 2014, 30.3% of high school and 42.2% of middle school students had never smoked traditional cigarettes. Given the concern that significant rates of e-cigarette use by U.S. adolescents may have a negative effect on public health, further review of e-cigarette advertising, marketing, sales, and use among U.S. youth is warranted.

## Introduction

Electronic cigarettes (e-cigarettes) are battery-powered devices that deliver nicotine in the form of an aerosol [1]. E-cigarettes that are not marketed for therapeutic purposes are currently unregulated by the Food and Drug Administration (FDA). Concurrent with increasing rates of adult e-cigarette use [2,3], youth e-cigarette use has also risen. National data found increased e-cigarette use from 2011 and 2012, with 2.7% of middle school students and 10.0% of high school students reporting ever use. Potential concerns about youth e-cigarette use have been raised, including the negative impact of nicotine on adolescent brain development [4–9], the potential risk for nicotine addiction, and the possibility of initiation of traditional cigarette or other tobacco product use [4,10,11]. This study examines recent trends in the prevalence of e-cigarette use among youth in Florida from 2011 to 2014.

## Materials and Methods

Data are from the annual 2011–2014 Florida Youth Tobacco Survey (FYTS), a school-based, pencil-and-paper questionnaire given to Florida middle (grades 6–8) and high school (grades 9–12) students. FYTS is a cross-sectional, representative state sample based on a two-stage cluster probability sample design [12]. A random sample of public middle and high schools was selected. Within each selected school, a random sample of classrooms was selected; all students in selected classes were eligible to participate. Data were statistically weighted to represent state-level estimates using the study sampling frame from the U.S. Department of Education. Post-stratification weights were based on population totals for each combination of students’ grade, race, and gender. Yearly sample sizes ranged from 5,972 to 38,972 for middle school students and from 6,097 to 36,578 for high school students over the study period. Overall participation rates ranged from 73% to 83%. The study was reviewed and exempted as ongoing public health surveillance by the Florida Department of Health institutional review board.

We estimated the prevalence of ever and current use of e-cigarettes, traditional cigarettes, and both. Ever use of e-cigarettes was defined as an affirmative response to the item “Have you ever tried once using electronic cigarettes?” (yes/no); current use was defined as an affirmative response to the item “During the past 30 days have you used an electronic cigarette?” (yes/no). Ever use of traditional cigarettes (experimentation) was measured with the item “Have you ever tried cigarette smoking, even one or two puffs?” (yes/no) and defined as students who responded affirmatively. Current traditional cigarette use (past 30-day use) was measured with the item “During the past 30 days, on how many days did you smoke cigarettes?” (0, 1 or 2, 3 to 5, 6 to 9, 10 to 19, 20 to 29, all 30 days) and defined as reported use on 1 or more days.

We conducted analyses using Stata 13.1 with complex survey design estimators to account for sample weights. We conducted descriptive analyses of e-cigarette use by plotting yearly rates of ever and current use among middle and high school students separately. We conducted logistic regressions to examine the change in ever use, current use, and concurrent use of products over time. Logistic regressions were also used to examine current e-cigarette use among never smokers over time. To examine the trend in the prevalence of ever use and current use, we estimated a logistic regression model including survey year indicators with 2011 as the referent. The demographic variables grade, gender, and race/ethnicity were examined as potential control variables in the models and did not alter the findings. A t-test using a cluster robust standard error to account for intercorrelations was conducted to examine the difference between 2014 current traditional cigarette and e-cigarette use. T-tests (with Bonferroni correction to p-values as appropriate) were used to examine the differences in past 30-day use of e-cigarettes within each demographic subgroup (e.g., comparisons between grade 6 and 7, grade 7 and 8, grade 6 and 8); similarly, differences in past 30-day use of traditional cigarettes by demographics were examined. Statistically significant results were reported at *P*<0.05 or less.

## Results

### Trends among Middle School Students

**Fig 1:**
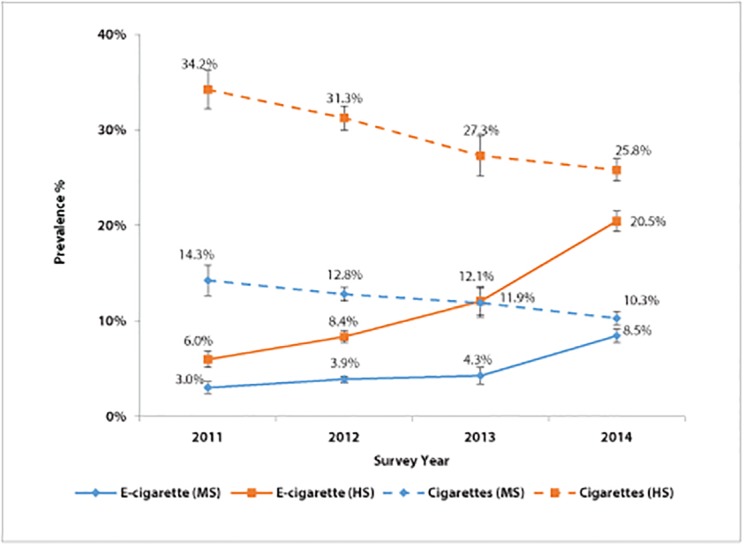
Ever electronic cigarette and traditional cigarette use among middle and high school students, by year—Florida Youth Tobacco Survey, 2011–2014. The largest increase in ever use of electronic cigarettes occurred between 2013 and 2014 for middle and high school students. MS = middle school. HS = high school.

**Fig 2:**
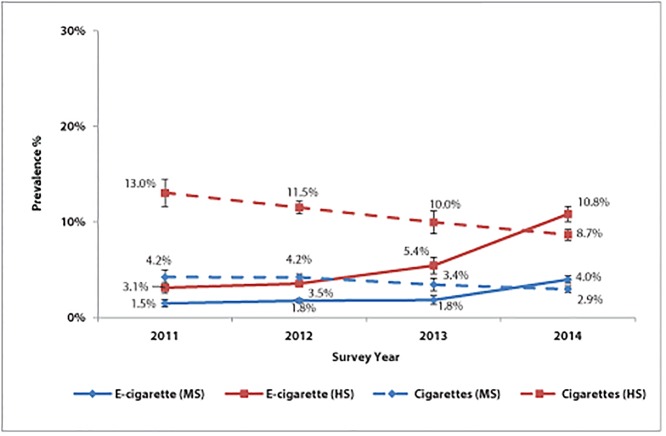
Past 30-day electronic cigarette and traditional cigarette use among middle and high school students, by year—Florida Youth Tobacco Survey, 2011–2014. The largest increase in past 30-day use of electronic cigarettes occurred between 2013 and 2014 for middle and high school students. MS = middle school. HS = high school.

Traditional cigarette and e-cigarette use among youth from 2011 to 2014 is shown in Figs 1 and 2. Logistic regression models indicate that, among middle school students, ever e-cigarette use increased threefold over the 4-year period (OR = 2.96, *P*<0.001, Table 1), from 3.0% in 2011 to 8.5% in 2014 (Fig 1). Current use (past 30-day use) also significantly increased over 4 years, from 2011 (1.5%) to 2014 (4.0%) (Fig 2, Table 1). Both ever and current use of traditional cigarettes declined from 2011 to 2014 (Figs 1 and 2, Table 1).

**Table 1:** Logistic regression models examining change over time in traditional cigarette use and e-cigarette use among Florida youth—Florida Youth Tobacco Survey, 2011–2014.

	Ever use traditional cigarettes	Past 30-day use traditional cigarettes	Ever use e-cigarettes	Past 30-day use e-cigarettes
	OR	OR	OR	OR
	(95% CI)	(95% CI)	(95% CI)	(95% CI)
Middle School				
Year 2011	*Reference*	*Reference*	*Reference*	*Reference*
Year 2012	0.88	0.99	1.30*	1.20
	(0.76–1.02)	(0.81–1.22)	(1.04–1.64)	(0.89–1.61)
Year 2013	0.81*	0.81	1.43*	1.24
	(0.67–0.99)	(0.62–1.05)	(1.06–1.93)	(0.86–1.80)
Year 2014	0.69 ***	0.69**	2.96***	2.77***
	(0.59–0.80)	(0.55–0.86)	(2.36–3.73)	(2.06–3.71)
High School				
Year 2011	*Reference*	*Reference*	*Reference*	*Reference*
Year 2012	0.87*	0.87	1.44 ***	1.14
	(0.79–0.97)	(0.75–1.00)	(1.21–1.70)	(0.93–1.41)
Year 2013	0.72***	0.74**	2.16***	1.79***
	(0.63–0.83)	(0.61–0.89)	(1.76–2.65)	(1.40–2.30)
Year 2014	0.67***	0.63***	4.04 ***	3.79***
	(0.60–0.74)	(0.55–0.73)	(3.44–4.76)	(3.09–4.64)

In 2014, 4.0% of middle school students reported past 30-day use of e-cigarettes, compared with 2.9% for current traditional cigarette use (*P*<0.001). Among middle school students in 2014, 42.2% of ever e-cigarette users reported never smoking traditional cigarettes, whereas 33.6% of current e-cigarette users reported currently smoking traditional cigarettes. Similar to trends among all middle school youth, logistic regression models indicate that past 30-day use of e-cigarettes among never smokers of traditional cigarettes was more than four times higher in 2014 (1.7%) than in 2011 (0.4%, OR = 4.62, *P*<0.01). Concurrent use of e-cigarettes and traditional cigarettes in the past 30 days was also higher in 2014 (1.3%) than in 2011 (0.8%, OR = 1.50, *P*<0.05). Among middle school students, current e-cigarette use was higher for students in grade 8 than grade 6 (*P*<0.001) and among students living in households with other tobacco users (*P*<0.001) or with e-cigarette users (*P*<0.001) (Table 2). Fewer black, non-Hispanic students were current e-cigarette users compared with white, non-Hispanic (*P*<0.001) and Hispanic students (*P*<0.001) (see Table 2).

**Table 2:** Current (past 30-day) use of electronic cigarettes and traditional cigarettes among middle and high school students, by demographic characteristics—Florida Youth Tobacco Survey, 2014.

Demographic Characteristic	Sample Size	Electronic Cigarette Use in Past 30 Days	Traditional Cigarette Use in Past 30 Days
		Weighted %	Weighted %
**Middle School**			
**Overall**	**36,993**		
**Grade**			
6	12,020	1.9%	1.7%^a^
7	12,520	3.6%^a^	2.6%^a^
8	12,012	5.8%^a^	3.7%^a^
**Gender**			
Female	18,430	3.6%	2.8%
Male	17,937	4.2%	3.0%
**Race/Ethnicity**			
White, non-Hispanic	17,645	4.3%^b^	3.0%
Black, non-Hispanic	5,930	3.1%^b^	2.6%
Hispanic	8,431	4.4%^b^	3.1%
Other race, non-Hispanic	3,455	3.9%^b^	3.2%
**Household Tobacco Use**			
Yes	13,353	8.0%^c^	5.5%^c^
No	18,975	1.7%^c^	1.2%^c^
**Household E-cigarette Use**			
Yes	4,180	16.4%^d^	8.5%^d^
No	28,004	2.3%^d^	1.9%^d^
**High School**			
**Overall**	**32,930**		
**Grade**			
9	9,944	8.8%^e^	6.0%^a^
10	8,525	10.7%^e^	7.4%^a^
11	7,608	11.6%^e^	8.8%^a^
12	6,403	11.8%^e^	11.6%^a^
**Gender**			
Female	16,330	8.7%^f^	7.6%^f^
Male	16,084	12.6%^f^	9.4%^f^
**Race/Ethnicity**			
White, non-Hispanic	16,853	14.2%^i^	10.6%^g^
Black, non-Hispanic	5,311	4.0%^i^	3.6%^g^
Hispanic	7,531	11.1%^i^	9.0%^g^
Other race, non-Hispanic	2,596	11.2%^i^	11.2%^g^
**Household Tobacco Use**			
Yes	12,951	18.6%^c^	14.4%^c^
No	17,170	5.7%^c^	4.3%^c^
**Household E-cigarette Use**			
Yes	4,233	33.0%^d^	21.7%^d^
No	25,734	7.3%^d^	6.1%^d^

^a^ Statistically significant differences between each grade level (*P*<0.01).

^b^ Statistically significant difference between white, non-Hispanic and black, non-Hispanic and also between black, non-Hispanic and Hispanic (*P*<0.01). “Other race” category includes Asian, American Indian or Alaskan Native, Native Hawaiian or other Pacific Islander, and Other race.

^c^ Statistically significant difference (*P*<0.001). Use defined as cigarettes, cigars, chewing tobacco, snuff, dip, or hookah.

^d^ Statistically significant difference (*P*<0.001). Use defined as electronic cigarettes regardless of other tobacco product use.

^e^ Statistically significant difference between grade 9 and each other grade level (grade 10, grade 11, and grade 12) (*P*<0.001). No differences between grade 10, grade 11, and grade 12.

^f^ Statistically significant difference (*P*<0.001).

^g^ Statistically significant difference between all groups (*P*<0.02), except white, non-Hispanic and Other (not significant).

^h^ Statistically significant differences across all groups (*P*<0.01), except Hispanics and Other (not significant).

### Trends among High School Students

Among high school students, ever e-cigarette use increased fourfold during the study period (OR = 4.04, *P*<0.001, Table 1). Ever use increased from 6.0% in 2011 to 20.5% in 2014, with the largest increase occurring between 2013 and 2014 (8.4 percentage points) (Fig 1). Current e-cigarette use also increased from 2011 (3.1%) to 2014 (10.8%) (Fig 2, Table 1). In comparison, 8.7% of high school students were current traditional cigarette users in 2014 (significantly different from 2014 e-cigarette use at *P*<0.001). Both ever and current use of traditional cigarettes declined over the study period (Figs 1 and 2, Table 1).

Among high school ever e-cigarette users in 2014, 30.3% reported never smoking traditional cigarettes. Among high school current e-cigarette users, 43.1% also reported current traditional cigarette smoking. Among high-school never smokers, models indicate that past 30-day use of e-cigarettes increased sixfold between 2011 (0.6%, OR = 6.33, *P*<0.01) and 2014 (3.7%). Concurrent use of e-cigarettes and traditional cigarettes in the past 30 days was also higher in 2014 (4.5%) than in 2011 (2.4%, OR = 1.96, *P*<0.01).

Findings for high school student demographic subgroups were similar in pattern to those found among middle school students with notably larger differences by gender and race/ethnicity (Table 2). Males reported current e-cigarette use more often than females (*P*<0.001). Fewer black, non-Hispanic students were current e-cigarette users compared with white, non-Hispanic and Hispanic students (*P*<0.001) (Table 2).

## Discussion

Experimentation with and past 30-day use of e-cigarettes increased threefold among Florida middle and high school students from 2011 to 2014. Between 2013 and 2014, current e-cigarette use doubled among middle and high school students in Florida. An estimated 105,900 Florida youth in grades 6 through 12 used one or more e-cigarettes in the past 30 days in 2014. The 2014 prevalence rates for current e-cigarette use are unprecedented, exceeding traditional cigarette use among middle and high school students. In 2014, 30.3% of high school students and 42.2% of middle school students who experimented with e-cigarettes had never tried traditional cigarettes. More males than females reported past 30-day e-cigarette use, while fewer black, non-Hispanic students reported use compared with white, non-Hispanic and Hispanic students. In contrast to rising e-cigarette experimentation among Florida youth, both ever and current use of traditional cigarettes declined steadily over the study period for middle and high school students.

Limitations of the data include the use of cross-sectional surveys, which cannot determine the extent to which each type of product use influences the other. Also, these are self-reported behaviors, which may include inaccuracies and cannot be validated. In addition, ever and current use are not mutually exclusive as assessed and do not fully capture patterns of use. Past 30-day use of products does not reflect regular or daily use, and our study does not assess whether patterns of use differ between youth cigarette smoking and e-cigarette use. However, these limitations were stable across years and do not affect study trends over time.

This descriptive study is the first to document the rise in statewide e-cigarette prevalence rates in recent years, which tripled between 2011 and 2014 and resulted in an unprecedented number of Florida youth using e-cigarettes. Given FDA’s concern that significant rates of e-cigarette use by U.S. adolescents could have a negative effect on public health [13], this study underscores the need for rapid regulatory action to prevent advertising, marketing, sales, and use of e-cigarettes among youth.

## Supporting Information

S1 File The file includes variables that were used to conduct the analysis reported in this study. (XLSX)
